# How We Treat Wounds To-Day

**Published:** 1886-11

**Authors:** 


HOW WE TREAT WOUNDS TO-DAY. *A Treatise on the Subject of Antiseptic Surgery which can be understood by beginners. By* ROBERT T. MORRIS, M.D. *Second Edition; pp. 165. New York:* G. P. PUTNAM'S SONS. *Chicago:* A. C. MCCLURG & CO., 1886.

This book deserves the enthusiastic reception which it has received. The only important change in the second edition is an attempt to explain the success of Lawson Tait's abdominal surgery without the use of antiseptics. Everyone who reads this attempted explanation will probable form an opinion of his own regarding the attempted explanation. The conclusion of the author's argument is, that while a few men seem able to discard ordinary so-called antiseptic measures without bad results, the great majority cannot do so. He also claims that those who decry careful scientific antisepsis are men who do not understand it.

Complete directions are given for the antiseptic treatment of all classes of wounds, from a simple incised wound to extensive burns and laparotomies, and the steps of the operations are so carefully and fully explained that even beginners can apply them in actual practice. If the principles proclaimed in this little book were rigidly carried out by all surgeons there would be much less inflammation and pus in wounds and fewer deaths both in hospital and private practice. The little volume is destined to do much more good in the world than many a larger one.

